# Convergence and divergence in gene expression among natural populations exposed to pollution

**DOI:** 10.1186/1471-2164-8-108

**Published:** 2007-04-25

**Authors:** Marla A Fisher, Marjorie F Oleksiak

**Affiliations:** 1 Department of Biology, University of Hawai'i, Hilo, 200 W. Kawili St., Hilo, HI 96720, USA; 2 Rosenstiel School of Marine & Atmospheric Sciences, University of Miami, 4600 Rickenbacker Cswy, Miami, FL 33149 USA; 3 Department of Environmental and Molecular Toxicology, North Carolina State University, Campus Box 7633, Raleigh, NC 27695-7633 USA

## Abstract

**Background:**

Natural populations of the teleost fish *Fundulus heteroclitus *tolerate a broad range of environmental conditions including temperature, salinity, hypoxia and chemical pollutants. Strikingly, populations of *Fundulus *inhabit and have adapted to highly polluted Superfund sites that are contaminated with persistent toxic chemicals. These natural populations provide a foundation to discover critical gene pathways that have evolved in a complex natural environment in response to environmental stressors.

**Results:**

We used *Fundulus *cDNA arrays to compare metabolic gene expression patterns in the brains of individuals among nine populations: three independent, polluted Superfund populations and two genetically similar, reference populations for each Superfund population. We found that up to 17% of metabolic genes have evolved adaptive changes in gene expression in these Superfund populations. Among these genes, two (1.2%) show a conserved response among three polluted populations, suggesting common, independently evolved mechanisms for adaptation to environmental pollution in these natural populations.

**Conclusion:**

Significant differences among individuals between polluted and reference populations, statistical analyses indicating shared adaptive changes among the Superfund populations, and lack of reduction in gene expression variation suggest that common mechanisms of adaptive resistance to anthropogenic pollutants have evolved independently in multiple *Fundulus *populations. Among three independent, Superfund populations, two genes have a common response indicating that high selective pressures may favor specific responses.

## Background

Many natural populations are continuously exposed to chemical stressors. One indication of this is that in 2004, over 4.24 billion pounds of industrial chemicals were disposed or released to the environment by facilities required to report to the EPA, including 576 million pounds of air emissions and 210 million pounds of surface water releases [1]. What are the biological consequences of chronic exposure to environmental pollution? We addressed this question using natural populations of the teleost *Fundulus heteroclitus *that inhabit and have adapted to highly polluted Superfund sites [2-5]. These populations are exposed to some of the highest concentrations of aromatic hydrocarbon pollutants of any vertebrate species [6]. Compelling evidence for adaptation in these populations is that embryos from the polluted sites are resistant to the toxic effects of the contaminated sediments while reference embryos are not [2-5]. Both first and second generation embryos from Superfund sites at New Bedford Harbor and Elizabeth River and first generation embryos from depurated Newark Bay fish are resistant, suggesting that differential survival is due to genetic adaptation rather than physiological induction. The central question we asked is, what differences in metabolic gene expression appear to be evolutionarily important within and among populations subject to anthropogenic stress?

To address this question we examined brain gene expression in individuals from three independent populations of *Fundulus *that inhabit and have adapted to highly polluted Superfund sites. The Superfund sites are highly contaminated with aromatic hydrocarbons, including polychlorinated biphenyls (PCBs), dibenzo-*p*-dioxins, and polycyclic aromatic hydrocarbons (PAHs) among other contaminants [7-9]. Gene expression was measured in field-caught fish raised for one year in common re-circulating tanks which removes physiological differences among populations induced by temperature, water quality, feeding, and other environmental factors [10]. Although many pollutants have long residual body-times, one year depuration should eliminate most pollutants (highly chlorinated PCB half-lives in trout are 70 days [11], dioxin half-life in *Fundulus *is <60 hours [12]). We assume that differences found after this common gardening are predominantly genetic rather than physiological.

We used a metabolic array since exposure to stress is likely to affect metabolic gene expression due to the additional energy needed to protect against harmful effects. Metabolic gene expression was examined in brains since brain tissue is metabolically active and may be highly susceptible to lipophilic pollutants: high energy demands, high lipid content, low antioxidant levels, reactive microglial cells, and inability to replicate neurons make it vulnerable to pollution and resulting oxidative stress [13,14].

Multiple *Fundulus *populations that have independently evolved adaptive resistance to complex suites of pollutants provide independent contrasts for identifying genes involved in adaptation. We compared brain gene expression levels in common gardened *Fundulus *heteroclitus from three independent, resistant Superfund sites to individuals from six nearby reference populations. We report significant differences in gene expression that were unique to each polluted population or shared among two polluted populations as well as conserved responses in gene expression among all three Superfund populations.

## Results

### Population genetics and experimental design

We found large amounts of significant variation in gene expression among individuals within each of nine populations (38–61% of genes differed among individuals within populations, p < 0.01). These results are similar to previous studies of *Fundulus *metabolic gene expression [15-17]. Using a mixed model to test for differences between populations but without considering geographic distances between populations (relatedness) or whether a population is polluted, up to 25% of the genes have significant differences in expression among any two of the nine populations (p < 0.01). On average 12% of the genes differ between populations. Two reference populations (in CT and NJ) spanning a steep transition zone between northern and southern populations of *Fundulus *[18,19] exhibit the most differences. Because most of the variation in gene expression among populations is a function of the variation within populations (r^2^: 0.63–0.83; see Additional file 1), these differences are consistent with a neutral or nearly-neutral model of evolution [20].

**Figure 1:**
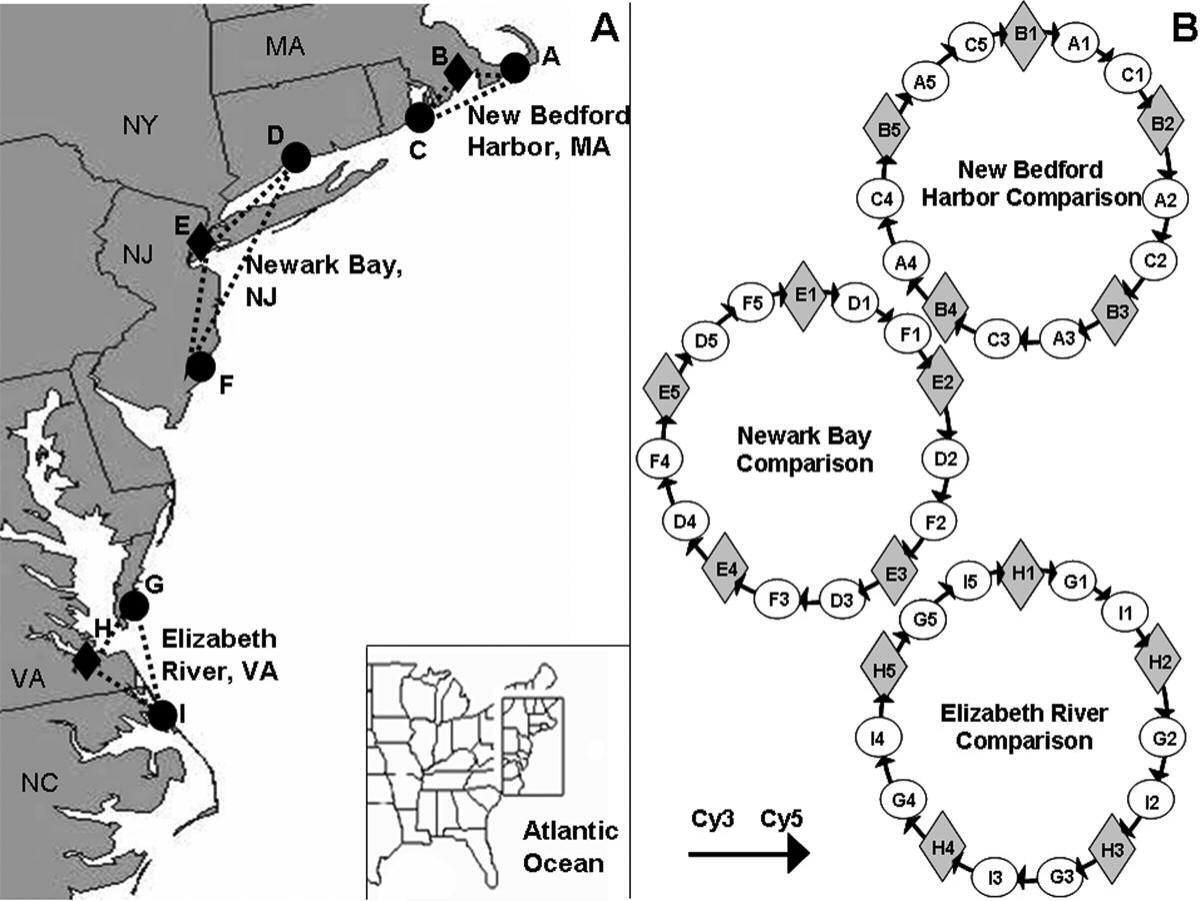
Sampling and Experimental Design. **A) **Map of sampling sites, USA, showing polluted Superfund Sites (diamonds, B, E and H) and reference sites (circles). Dashed triangles show the population comparisons illustrated in (B). Superfund sites have text labels. Site names, latitudes and longitudes are in Additional file 2. **B) **Loop design showing 5 individuals (1–5) from a polluted Superfund population (shaded diamond) hybridized with 5 individuals from each of two reference sites (circles). This experiment used 3 loops and 15 individuals per loop for a total of 45 individuals hybridized to 45 arrays. Each individual is labeled with both Cy3 and Cy5 dyes. Spots are replicated 4 times per array.

With the large number of differences in gene expression among individuals within a population, one should expect differences between genetically distinct populations. These differences are not necessarily due to evolution by natural selection since the variation in expression does not necessarily affect a biologically important phenotype. To distinguish biologically important differences, those evolving by natural selection, from among the many genes exhibiting significant differences within and among populations, we specifically tested whether gene expression in a Superfund population is statistically different from the two reference populations (Fig. 1, see Additional file 2). These three populations have similar genetic distances from each other such that the Superfund population and the two reference populations share similar numbers of neutral alleles and have similar differences in allele frequency [18]. Without the effect of natural selection, the combined variation in the two reference populations should be greater than the variation between the Superfund population and each reference population. In an analysis of variance (ANOVA), the variation between the polluted *versus *two reference populations will be significant if it is greater than the average variation within the polluted population and the combined reference populations. If the variation is significant, it exceeds the expectation of neutral evolutionary models [20] and thus is consistent with evolution by natural selection. Traits evolving by natural selection affect biologically important traits. Thus, this analysis of variance provides evidence for which gene expression changes are of functional significance.

We also asked which gene expression levels were significantly different in all three polluted populations compared to all six reference populations. Since there is as much genetic distance among populations within these two groups (polluted or reference) as there is between groups (polluted *versus *reference), the variation in gene expression due to random drift is accounted for in the ANOVA. Thus, similar to the separate comparisons with the three Superfund populations, the genetic differences in gene expression most likely represent evolution by natural selection and in these comparisons, adaptation to chronic exposure to pollution.

### Differences in brain gene expression between polluted and paired reference populations

A linear mixed model was used to test for differences between Superfund populations *versus *the two surrounding reference sites. We made three comparisons: each Superfund population *versus *two reference populations (Fig. 1). For the most northern Superfund population, New Bedford Harbor, MA, 37 genes are significantly differentially expressed compared to two reference populations (17%, p < 0.01). For the Superfund site in Newark Bay, NJ, 27 genes are differentially expressed (13%, p < 0.01). For the most southern Superfund site, Elizabeth River, VA, 13 genes are significantly different (5%, p < 0.01). These numbers are greater than the 2 genes expected by chance (i.e. 1% of 200 analyzed genes). After a Bonferroni correction for multiple comparisons, 9, 6 and 1 genes are significantly different in New Bedford Harbor, Newark Bay and Elizabeth River populations, respectively [see Additional file 3]. We did not find greater inter-individual variation in gene expression in the Elizabeth River and surrounding reference populations. Thus, the failure to detect as many differences in expression in the Elizabeth River population compared to the New Bedford Harbor and Newark Bay populations is not due to greater individual variation.

### Common gene expression differences among all polluted populations

**Figure 2:**
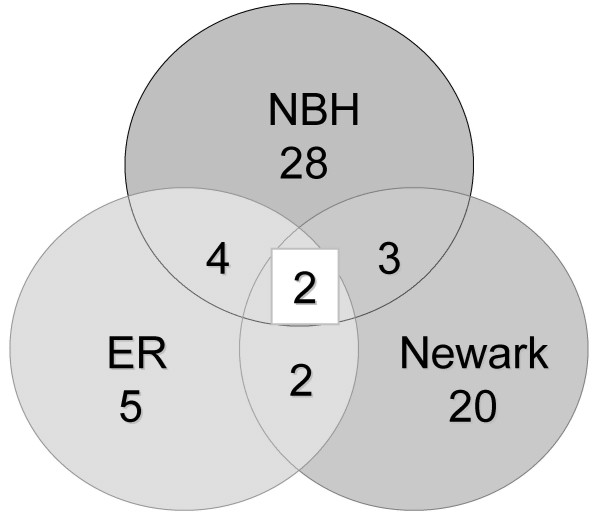
Venn Diagram showing numbers of significant genes (p < 0.01) for each polluted *versus *reference sites comparison. Two genes are significantly different in all three polluted populations compared to their respective reference populations. NBH = New Bedford Harbor comparison, Newark = Newark Bay comparison, ER = Elizabeth River comparison.

The Venn diagram summarizes significant genes (p = 0.01) for each comparison of a Superfund population to two reference populations (Fig. 2). The New Bedford Harbor population had 5 genes in common with the Newark Bay population and 6 genes in common with the Elizabeth River population. The Newark Bay population had 4 genes in common with the Elizabeth River population. Two genes are common to all 3 populations, a NADH-ubiquinone oxidoreductase AGGG subunit precursor (NDUB2 or NDUBF2), a subunit in oxidative phosphorylation complex I, and thioredoxin, a general protein disulfide oxidoreductase.

**Figure 3:**
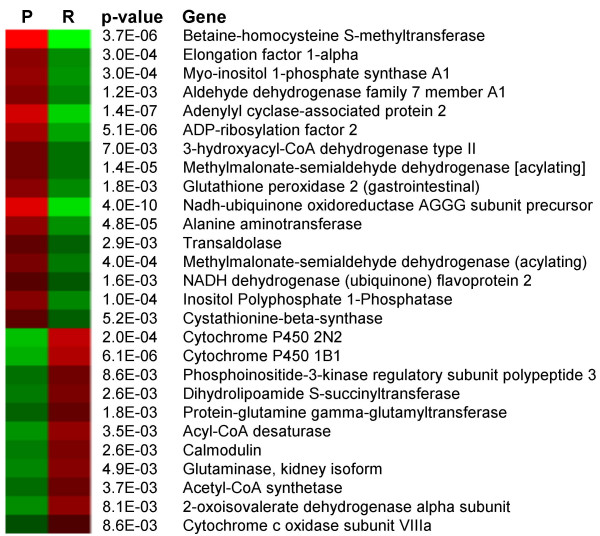
All polluted sites *versus *all reference sites. Heat map, p-values, and gene expression increased (red) and decreased (green) in a comparison of all polluted sites (P) *versus *all reference sites (R).

We statistically compared brain gene expression patterns among individuals from all three polluted populations (New Bedford Harbor, Newark Bay, and Elizabeth River) to individuals from all six reference populations to determine if there were gene expression changes due to common pollution stress. Analyses indicate that 17% (27/170) of the genes were more similar among polluted populations and significantly different from reference populations at p = 0.01 (Fig. 3). Sixteen genes were more highly expressed and eleven genes were less expressed. After a Bonferroni correction, eleven genes remain significant.

Table 1 shows the p-values associated with these 27 statistically significant genes between all polluted populations *versus *all reference populations contrasted with p-values from separate population comparisons of a polluted population *versus *two reference populations. NDUB2 is consistently differentially expressed at p < 0.01 in the four separate comparisons: the three separate comparisons of each Superfund site *versus *surrounding reference sites and the comparison of all polluted *versus *all reference populations. At p < 0.05, CYP1B1 also is consistently differentially expressed in all four comparisons.

## Discussion

### Metabolic gene expression differences in polluted populations

We used natural populations of *Fundulus *that inhabit and have adapted to highly polluted Superfund sites to identify gene expression changes involved in adaptation to pollution. Expression of 17% of the metabolic genes examined differed between the New Bedford Harbor, MA Superfund population compared to two reference populations, 12% differed in the Newark Bay, NJ comparison, and 5% differed in the Elizabeth River comparison. Genes contributing to phase I and phase II biotransformation of xenobiotics are likely candidates for adaptation at the physiological or genetic level in pollution-impacted populations. Transcripts for the phase I enzymes CYP2N2 and CYP1B1 were less expressed in the New Bedford Harbor population and CYP2N2 expression also was lower in the Newark Bay population. CYP1A induction is important in many toxic responses to dioxin-like compounds although it is refractory to induction in individuals from all of these polluted populations [2,3,21]. Not unexpectedly, its expression did not differ in these un-induced individuals.

Several gene expression changes are consistent with toxic effects from exposure to dioxin-like contaminants. For instance, expression of many genes involved in oxidative phosphorylation differed in the three polluted populations, as might be expected since dioxin affects the respiratory mitochondrial electron chain [22]. Additionally, in response to an increase in reactive oxygen species caused by leakage from the electron transport chain or directly *via *phase I and II metabolism, there is an increase in glutathione S-transferase A and glutathione peroxidase expression, as seen in other studies [23,24]. The expression of superoxide dismutases (Cu-Zn and Mn), and glutathione S-transferases mu 3 and mu 5 did not differ. Interestingly, all three Superfund populations had differences in the expression of fatty acid binding protein genes which may be central regulators in inflammatory and metabolic signaling [25].

### Common significant genes among polluted populations

An important question for adaptation in general is whether common solutions to environmental stress evolve. Recent transcriptional studies suggest multiple mechanisms to a biological end [26,27]. In *Drosophila*, the number of evolved responses depends on selection strength: altered expression of different cytochrome P450s (CYP) develops in response to DDT in laboratory *Drosophila*, yet in resistant field isolates, over-expression of CYP6g1 predominates [28] (but see [29]). One approach to address this question is to discern significant changes in expression shared among Superfund populations (Fig. 2). Two genes are common in all 3 populations, the NADH-ubiquinone oxidoreductase AGGG subunit precursor (NDUB2), a subunit in oxidative phosphorylation complex I, and thioredoxin, a general protein disulfide oxidoreductase. However only NDUB2 has a consistent pattern with significantly more highly expressed in the Superfund populations. Thioredoxin expression was greater in one Superfund population but decreased in the other two, suggesting that the expression of this gene is evolutionarily labile.

A statistically more powerful test of a shared adaptive response in gene expression is to compare all three polluted populations to all six reference populations. In these analyses, expression levels of 17% of the genes examined were statistically significantly different in the polluted populations compared to reference populations. These differentially expressed genes suggest a shared response to pollution stress in the independent polluted populations. However, by comparing significant expression among polluted populations, many of these differences in gene expression appear to be driven by only one or two of the Superfund populations (Table 1) and might reflect the different pollutants at each site. Only NDUB2 is consistently differentially expressed at p < 0.01 in the three separate comparisons of each Superfund site *versus *surrounding reference sites and also in the comparison of all polluted *versus *all reference populations. Thus its altered expression is unlikely to be due to a type I error. If we are interested in defining the limited number of genes with a common response among all polluted populations, we are concerned with type II error (acceptance of a false null hypothesis) rather than type I error. To better control for type II error we use a less stringent significance level of p < 0.05 and stipulate that direction of expression is consistent (always up or down in polluted *versus *reference populations). At this significance level, CYP1B1 also is consistently differentially expressed in all four comparisons. Thus, in the comparisons of each superfund site *versus *its two reference sites and among the three polluted *versus *six reference populations comparison, two of the 170 genes (1.2%) appear to have shared derived changes in gene expression in response to anthropogenic pollution.

### Evolutionarily important adaptive genes

We suggest that these two genes are of critical importance in stress response and are involved in resistance to pollution stress. For instance, NDUB2 is part of complex I in oxidative phosphorylation. Defects in this complex have been linked to Parkinson's disease and other genetic metabolic diseases, and modulation of complex 1 activity can generate oxidative stress [30,31]. Although we analyzed 27/46 subunits of NADH-ubiquinone oxidoreductase, only NDUB2 was significantly more highly expressed in polluted populations, suggesting that its transcription may be limiting. This is surprising since transcriptional activators and coactivators such as nuclear respiratory factors NRF1 and 2 co-regulate many nuclear encoded, mitochondrial genes [32], and expression of NDUB2 was positively correlated with many, but not all, complex I subunits, suggesting a common transcriptional regulation. If there is a common transcriptional regulation for NDUB2, then one would expect many complex I genes to be significantly altered. Although we used 45 individuals, a greater sample size might have identified significant differences in expression for more complex I genes.

CYP1B1 has a physiological induced responsiveness to exposure to dioxin-like compounds [33] found in these Superfund sites. While CYP1B1 is usually induced [34], we found significantly lower expression levels. Since CYP1B1 activates chemically diverse procarcinogens [35], our analyses suggest that decreased expression may be a protective genetic response to chronic pollutant exposure. These two genes, NDUB2 and CYP1B1, are not significantly correlated (p > 0.2) suggesting that the variation in their expression is not due to a single *trans*-acting factor. The observation that consistent differences in these two genes have evolved independently in these three populations suggests that the changes in gene expression are evolutionarily important and thus functionally important.

If these Superfund populations were similar to breeding selection experiments, we would expect a decrease in variance compared to reference populations. Among individuals in Superfund populations, the average variation in gene expression is 2.3 ± 1.8 and in the six reference populations it is 2.2 ± 1.6, and the correlation of variances among genes is 97%. Statistically, there is a homogeneity of variance among these two groups. Why would the variances be similar? Pollution is a relatively recent event compared to the evolutionary history of this species. These polluted populations clearly have adaptive differences in survival and development that are maintained in the first and second generations [2-5]. Yet, we find no reduction in the variation in gene expression in polluted populations. Since most of the variation in gene expression is thought to be genetically based [15,36,37], maintenance of variation in gene expression most likely represents steady influx of alternative alleles by migration. Migration in this species, although empirically low, is enough to minimize genetic difference among populations in Chesapeake Bay [38], and among populations greater than 50 km apart in Georgia [18]. The maintenance of variation for gene expression in populations subjected to significant selection suggests that outbreeding and migration have mitigated the loss of genetic variation and allelic variation has been maintained. Consequently, the shared selective differences in gene expression are both biologically important and represent an advantageous and superior response among the many options.

## Conclusion

Independent *Fundulus *populations provide the opportunity to discover adaptive responses to pollution evolved in a complex natural environment. In each of the Superfund populations 5 to 17% of genes are differentially expressed, yet only two genes (1.2%) have a consistent difference in expression among all polluted sites. Consistent significant differences in gene expression between polluted and reference populations, statistical analyses that indicate evolved adaptive changes, and lack of reduction in variation in gene expression suggest that multiple *Fundulus *populations have independently evolved common mechanisms of adaptive resistance to complex suites of pollutants. Integrating an evolutionary population genetics approach with an environmental toxicology study allowed us to differentiate genes that vary due to chronic pollutant exposure from those that vary due to random genetic drift. The shared response of two genes among these three independent populations supports the idea that high selective pressures may favor similar responses.

## Methods

### Field fish collection, care and sampling

*Fundulus heteroclitus *were collected by minnow trap from three Superfund Sites (New Bedford Harbor, MA; Newark Bay, NJ; Elizabeth River, VA), and six reference populations where pairs of these reference sites were located north and south of each polluted site (Sandwich, MA and Pt. Judith, RI for New Bedford Harbor, MA; Tuckerton NJ and Clinton, CT for Newark, NJ: and Magnatha, VA and Manteo, NC for Elizabeth River, VA; Fig. 1). Fish were kept in a common re-circulating aquarium system with controlled temperature and salinity of 20°C, 15 ppt salinity for one year before experiments. Effluent from tanks was passed through an activated charcoal filter and 20% water changes were performed weekly. Tanks were cleaned and fish checked for health status on a daily basis. Fish were fed once daily a 33% mixture of brine shrimp flake, blood meal flake and *Spirulina *flake (FOD, Aquatic Ecosystems). Prior to sampling, fish were subjected to pseudo-winter (6:18, light: dark cycle) for four to six weeks, then maintained for a minimum of 6 weeks with a light cycle of 16:8, light:dark. After the pseudo-winter, *Fundulus *came into reproductive condition and spawned, and reproductive tissues regressed. The reproductive tissues were in regression when fish were assayed.

Populations and individuals within a population were chosen randomly for sacrifice and sampled in the morning and early afternoon for two consecutive days in order to minimize physiological changes due to diurnal cycles. Five fish from each of 9 populations were sacrificed by cervical dislocation. Fish were mixed sex and ranged in weight, with an average weight of 7.28 ± 0.33 g. Neither sex nor weight were significant variables in the mixed models discussed below. Dissected organs, including brain, for subsequent RNA extraction were immediately frozen in a dry ice/ethanol bath and stored at -80°C. This experiment was performed according to an approved Institutional Animal Care and Use Committee at North Carolina State University.

### RNA extraction, amplification, hybridization, scanning

Total RNA was isolated from each brain using a guanidinium thiocyanate buffer [39] followed by purification using the Qiagen RNeasy Mini kit (Qiagen Inc., Valencia, CA, USA) according to the manufacturer's protocols. Purified RNA was quantified spectrophotometrically, and RNA quality was assessed by gel electrophoresis. RNA for hybridization was prepared by one round of amplification (aRNA) using Ambion Amino Allyl MessageAmp aRNA Kit to form copy template RNA by T7 amplification. Amino-allyl UTP was incorporated into targets during T7 transcription, and resulting amino-allyl aRNA was coupled to Cy3 and Cy5 dyes (Amersham Biosciences, Piscataway, NJ, USA).

Labeled aRNA samples (5 pmol/ul) were hybridized to slides in 12 ul of hybridization buffer (50% formamide buffer, 5× SSPE, 1% sodium dodecyl sulfate, 0.2 mg/ml bovine serum albumin, 1 mg/ml denatured salmon sperm DNA (Sigma), and 1 mg/ml RNAse free poly(A) RNA (Sigma) for 42.5 hours at 42°C. Slides were prepared by blocking according to the manufacturer's recommendations with an additional treatment of 66 mM sodium borohydride to minimize background autofluorescence [40]. After hybridization, non-specifically bound probe was washed off with SSC and the slides were spun dry and scanned using a ScanArray Express 4000 (Perkin Elmer). Scanner settings used for analysis were 90% laser power for Cy3, 80% laser power for Cy5 and photomultiplier tube (PMT) setting at 70%. Resulting 16 bit Tiff Images were quantified using IconoClust^® ^(CLONDIAG, Jena, Germany) spotfinding software.

### Metabolic arrays

Amplified cDNA sequences for 384 metabolic genes from *Fundulus heteroclitus *heart and liver libraries were spotted onto CodeLink activated slides (GE Healthcare, Piscataway, NJ) at the University of Miami core microarray facility (slides were the kind gift of D. Crawford). Each slide contained 4 spatially separated arrays, and each array contained 4 replicates of 384 spots (genes) including controls. Printed cDNAs encode essential proteins for cellular metabolism based on KEGG (Kyoto Encyclopedia of Genes and Genomes). Sequence information, annotation and gene ontology are available for *Fundulus *on the FunnyBase website [41].

Of these 384 genes, not all genes were analyzed. Unanalyzed spots included negative controls (random genomic amplification or *Ctenophore *specific cDNAs) and genes that either saturated the photomultiplier tube or had signals less than the negative controls. The number of genes examined were 214, 216, and 248 for the New Bedford Harbor, Newark Bay, and Elizabeth River comparisons, respectively, and the analysis combining all locations (9 populations) used 170 common genes.

### Experimental design

A loop design was used for the microarray hybridizations where each sample is hybridized to 2 arrays using both Cy3 and Cy5 labeled fluorophores [42]. In this experiment, each loop consisted of Cy3 and Cy5 labeled brain aRNA from 5 individuals from a polluted site (P) hybridized to Cy3 and Cy5 labeled brain aRNA from 5 individuals from each of 2 adjacent reference sites (R1 and R2), for a total among the 3 loops of 45 individuals hybridized to 45 microarrays. Each array had different combinations of individuals, and each loop formed was P1→ R1.1→ R2.1→ P2 → R1.2-→ R2.2→ P3→ R1.3→ R2.3→ P4→ R1.4→ R2.4→ P5 → R1.5→ R2.5→ P1 where each arrow represents a separate hybridization (array) with the individual at the base of the arrow labeled with Cy3 and the individual at the head of the arrow labeled with Cy5 (Fig. 1).

### Statistical analysis

Log_2 _measures of gene expression were normalized using a linear mixed model in SAS (JMP v6.0.0 with a microarray platform beta-version in SAS v9.1.3) to remove the effects of dye (fixed effect) and array (random effect) following a joint regional and spatial Lowess transformation in MAANOVA Version 0.98.8 for R to account for both intensity and spatial bias [43]. The model was of the form y_ij _= μ + A_i _+ D_j _+ (AxD)_ij _+ ε_ij_, where, y_ij _is the signal from the i^th ^array with dye j, μ is the sample mean, A_i _and D_j _are the overall variation in arrays (1–15) and dyes (Cy3 and Cy5), (AxD)_ij _is the array × dye interaction and ε_ij _is the stochastic error [44,45]. Residuals from this model were used for gene-by-gene analyses (below). For all loops, the coefficients of variation (CV ± StdErr) averaged across all genes were relatively low: 2.5% ± 0.28%, 4.75% ± 0.52%, 3.0 % ± 0.50% for New Bedford Harbor, Newark Bay, and Elizabeth River comparisons, respectively.

Normalized data were modeled by residual maximum likelihood on a gene-by-gene basis using a linear mixed model in SAS using PROC MIXED. To test for a treatment effect (effect of chronic exposure to pollution), normalized data (residuals from the mixed model normalization) were modeled using treatment, population nested in treatment and dye as fixed effects and array and spot nested in array as random effects according to the model r_ijkgm _= μ + A_i _+ D_j _+ T_k _+ P(T)_gk _+ S(A)_mi _+ ε_ijkgm_, where T_k _is the kth treatment (polluted or reference), P(T)_gk _is the gth population nested in treatment, and S(A)_mi _is the mth spot nested in array. To compare all nine sites (3 polluted and 6 reference populations hybridized across 3 separate loops), loop (L_l_), dye by loop interactions, and array nested in loop terms were added in the mixed model normalization according to the model y_ijl _= μ + A_i _+ D_j _+ (AxD)_ij _+ L_l _+ (DxL)_jl _+ A(L)_il _+ ε_ijl_. To compare all nine populations, we used population as a fixed effect in the gene-by-gene model according to the model r_ijgm _= μ + A_i _+ D_j _+ P_g_+ S(A)_mi _+ ε_ijgm_. To test for a treatment effect using all nine populations, loop, treatment × loop interactions, and population nested in loop × treatment interactions were incorporated as fixed effects into the gene-by-gene model according to the model r_ijklgm _= μ + A_i _+ D_j _+ T_k _+ L_l _+ (LxT)_lk_+ P(TxL)_gkl _+ S(A)_mi _+ ε_ijklgm_. To examine individual variation among locations, the mixed model gene-by-gene analysis used individual nested in population, population and dye as fixed effects according to the model r_ijghm _= μ + A_i _+ D_j _+ P_g _+ I(P)_gh _+ S(A)_mi _+ ε_ijghm_.

Mixed model analyses were performed for each loop or combined loop analysis, and we used a nominal p-value cut-off for significant genes of p < 0.01. Using this p-value reveals more genes that may differentially expressed but risks identifying genes that may be false positives. Genes identified as also being significant after false positives are reduced by a more stringent multiple comparison correction were analyzed using a Bonferroni correction (p = 0.05). Homogeneity of variance was assessed among all polluted *versus *reference populations using analysis of variance (ANOVA) and Brown-Forsythe's test. We also used Brown-Forsythe's test for homogeneity of variance among all populations. The heat map of differentially expressed genes using all nine populations was visualized using the Macintosh version [46] of TreeView [47]. The correlation matrix was performed using Pearson product-moment correlation in SAS.

## List of abbreviations used

polychlorinated biphenyls (PCBs), polycyclic aromatic hydrocarbons (PAHs), NADH-ubiquinone oxidoreductase AGGG subunit precursor (NDUB2), cytochrome P450 1B1 (CYP1B1), cytochrome P450 2N2 (CYP2N2), cytochrome P450 1A (CYP1A)

## Authors' contributions

MAF and MFO designed research; MAF performed research; MAF and MFO analyzed data; and MAF and MFO wrote the paper.

## Supplementary Material

Additional File 1 Variation among and within populations. Additional file 1 shows the relationship between variation in gene expression among and within populations. Click here for file

Additional File 2 Sites, latitude and longitude in this study. Additional file 2 provides full references for the sites depicted in Figure 1, including names, latitudes and longitudes. Click here for file

Additional File 3 Significant Differences in Expression. Additional file 3 lists genes and p-values that are significantly different in each Superfund *versu*s respective reference populations. Click here for file

## Figures and Tables

**Table 1:** Genes and p-values for differentially regulated genes.

**Gene**	**P *vs. *R**	**New Bedford Harbor**	**Newark Bay**	**Elizabeth River**
**Nadh-ubiquinone oxidoreductase AGGG subunit precursor**	**4.00E-10**	**2.20E-03**	**3.10E-06**	**1.00E-03**
Adenylyl cyclase-associated protein 2	**1.40E-07**	0.07	**1.80E-05**	**6.40E-03**
Betaine – homocysteine S-methyltransferase	**3.70E-06**	0.03	**4.60E-05**	0.06
ADP-ribosylation factor 2	**5.10E-06**	**1.00E-04**	0.02	0.31
Cytochrome P450 1B1	**6.10E-06**	**7.00E-04**	**1.00E-02**	0.02
Methylmalonate-semialdehyde dehydrogenase [acylating]	**1.40E-05**	**4.20E-06**	**1.50E-03**	0.91
Alanine aminotransferase	**4.80E-05**	0.03	**9.90E-06**	0.72
Inositol Polyphosphate 1-Phosphatase	**1.00E-04**	**1.00E-04**	0.03	0.28
Cytochrome P450 2N2	**2.00E-04**	**8.10E-06**	**3.10E-03**	0.58
Elongation factor 1-alpha	**3.00E-04**	**1.00E-02**	**1.80E-03**	0.42
Myo-inositol 1-phosphate synthase A1	**3.00E-04**	0.21	**2.00E-11**	0.66

Methylmalonate-semialdehyde dehydrogenase (acylating)	**4.00E-04**	**2.00E-10**	0.16	0.68
Aldehyde dehydrogenase family 7 member A1	**1.20E-03**	0.50	0.03	**2.90E-03**
NADH dehydrogenase (ubiquinone) flavoprotein 2	**1.60E-03**	**5.30E-03**	0.2	0.08
Glutathione peroxidase 2 (gastrointestinal)	**1.80E-03**	**7.00E-04**	0.5	0.05
Protein-glutamine gamma-glutamyltransferase	**1.80E-03**	**1.00E-03**	0.12	0.77
Calmodulin	**2.60E-03**	0.10	0.37	**1.00E-02**
Dihydrolipoamide S-succinyltransferase	**2.60E-03**	0.37	**8.40E-03**	0.21
Transaldolase	**2.90E-03**	**1.70E-03**	0.46	0.06
Acyl-CoA desaturase	**3.50E-03**	0.09	0.31	0.03
Acetyl-CoA synthetase	**3.70E-03**	0.02	0.04	0.62
Glutaminase, kidney isoform, mitochondrial precursor	**4.90E-03**	0.32	**1.00E-02**	0.25
Cystathionine-beta-synthase	**5.20E-03**	0.37	**8.60E-03**	0.10
3-hydroxyacyl-CoA dehydrogenase type II	**7.00E-03**	0.46	**1.80E-05**	0.12

Genes and p-values for differentially regulated genes between all polluted populations *versus *all reference populations (P *vs. *R) are contrasted with p-values from separate population comparisons of a polluted population *versus *two reference populations. One gene (in bold) that is significant between all polluted sites *versus *all reference sites is also significant in each population comparison. Significant gene values are in bold. Genes above the double line are also significant with a Bonferroni correction.
