# Supplementary material for: Convergence and divergence in gene expression among natural populations exposed to pollution
Source: BMC Genomics. 2007 Apr 25;8:108. doi: 10.1186/1471-2164-8-108 (PMC1868758; doi:10.1186/1471-2164-8-108)
Supplement: Additional File 1 — Variation among and within populations. Additional file 1 shows the relationship between variation in gene expression among and within populations. [file 1471-2164-8-108-S1.ppt]

## Slide 1
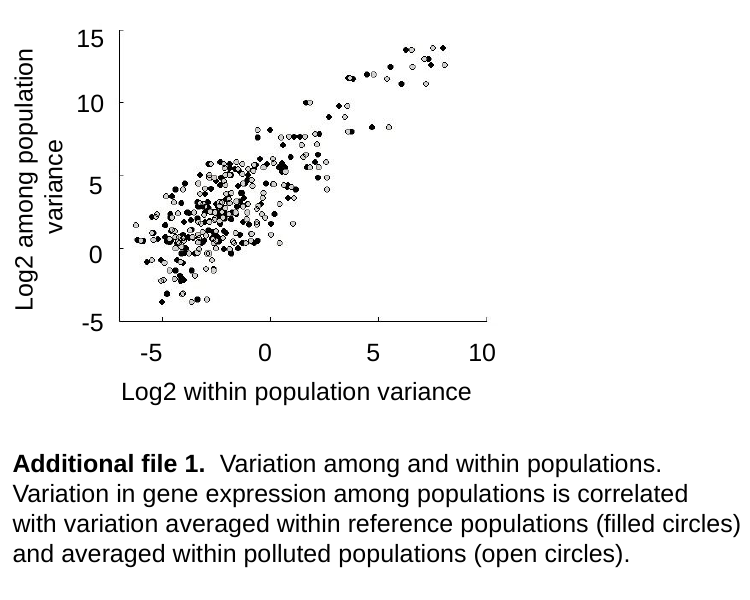

15
10
Log2 among population
 variance
5
0
-5
 0
-5
 5
 10
Log2 within population variance
Additional file 1. Variation among and within populations.
Variation in gene expression among populations is correlated
with variation averaged within reference populations (filled circles)
and averaged within polluted populations (open circles).
