# Supplementary material for: Convergence and divergence in gene expression among natural populations exposed to pollution
Source: BMC Genomics. 2007 Apr 25;8:108. doi: 10.1186/1471-2164-8-108 (PMC1868758; doi:10.1186/1471-2164-8-108)
Supplement: Additional File 2 — Sites, latitude and longitude in this study. Additional file 2 provides full references for the sites depicted in Figure 1, including names, latitudes and longitudes. [file 1471-2164-8-108-S2.doc]

**Additional file 2.** Sites, latitude and longitude in this study.

Superfund sites are designated as “SF”.

| Letter | Site | Latitude/ Longitude | Abbreviation^a^ |
| --- | --- | --- | --- |
| A | Sandwich, MA | 41^o^ 44.0'/ 70^o^ 23.0' | BSMA |
| B | New Bedford, MA (SF) | 41^o^ 39.9'/ 70^o^ 54.9' | NBHMA |
| C | Point Judith, RI | 41^o^ 21.7'/ 71^o^ 28.9' | PJRI |
| D | Clinton, CN | 41^o^ 15.3'/ 72^o^ 32.8' | CCN |
| E | Newark Bay, NJ (SF) | 40^o^ 41.2'/ 74^o^ 06.7' | NBNJ |
| F | Tuckerton, NJ | 39^o^ 32.2'/ 74^o^ 19.4' | TNJ |
| G | Magnatha, VA | 37^o^ 10.6'/ 75^o^ 56.5' | MVA |
| H | Elizabeth River, VA (SF) | 36^o^ 48.5'/ 76^o^ 17.7' | NVA |
| I | Manteo, NC | 35^o^ 53.8'/ 75^o^ 36.9' | RINC |

^a^Abbreviation as in Adams *et al.,* 2006
