# Supplementary material for: Convergence and divergence in gene expression among natural populations exposed to pollution
Source: BMC Genomics. 2007 Apr 25;8:108. doi: 10.1186/1471-2164-8-108 (PMC1868758; doi:10.1186/1471-2164-8-108)
Supplement: Additional File 3 — Significant Differences in Expression. Additional file 3 lists genes and p-values that are significantly different in each Superfund versus respective reference populations. [file 1471-2164-8-108-S3.doc]

**Additional file 3**. Significant Differences in Expression. Genes, p-values, least square means (LS Means) differences for differentially expressed genes in each of three comparisons of three polluted populations (NBH, Newark, ER) *versus* respective reference populations. Genes in bold are signfinicant after a Bonferroni correction. A gene with a positive least square means difference is more highly expressed in the polluted populations compared to the reference populations; a negative least square means difference value is expressed less in the polluted populations. NBH= New Bedford Harbor, Newark= Newark Bay, ER= Elizabeth River.

| **Polluted** | **Gene** | **P-value** | **LS Means** |
| --- | --- | --- | --- |
| **Site** |  |  | **Difference** |
| NBH | **Methylmalonate-semialdehyde dehydrogenase (acylating)** | 2.5E-10 | 0.13 |
|  | **Fatty acid-binding protein, retina** | 2.7E-08 | -0.13 |
|  | **Methylmalonate-semialdehyde dehydrogenase [acylating]** | 4.2E-06 | 0.08 |
|  | **Succinate dehydrogenase complex subunit C** | 6.0E-06 | 0.12 |
|  | **Cytochrome P450 2N2** | 8.1E-06 | -0.14 |
|  | **Phosphomannomutase 1** | 2.4E-05 | -0.21 |
|  | **ADP-ribosylation factor 2** | 5.4E-05 | 0.14 |
|  | **Ubiquinol-cytochrome c reductase core protein I** | 6.2E-05 | 0.15 |
|  | **Inositol Polyphosphate 1-Phosphatase** | 6.9E-05 | 0.08 |
|  | Fatty acid binding protein 7 brain | 8.8E-05 | -0.15 |
|  | Isocitrate dehydrogenase 2 | 9.6E-05 | 0.07 |
|  | Spi-1/PU.1 transcription factor | 9.8E-05 | -0.11 |
|  | Nucleoside diphosphate kinase A | 1.3E-04 | -0.11 |
|  | Aspartate aminotransferase mitochondrial precursor | 4.0E-04 | 0.12 |
|  | Isocitrate dehydrogenase 1 | 6.0E-04 | 0.10 |
|  | Cytochrome P450 1B1 | 7.0E-04 | -0.08 |
|  | Glutathione peroxidase 2 (gastrointestinal) | 7.0E-04 | 0.12 |
|  | Aldo-keto reductase family 1 member A1 | 7.0E-04 | 0.08 |
|  | Sodium/potassium-transporting ATPase alpha-3 chain | 8.0E-04 | -0.17 |
|  | ADP-ribosylarginine hydrolase | 8.0E-04 | 0.09 |
|  | Vacuolar ATP synthase subunit C | 8.0E-04 | 0.13 |
|  | Protein-glutamine gamma-glutamyltransferase | 1.0E-03 | -0.09 |
|  | ATP synthase H+ transporting mitochondrial F0 complex subunit b isoform 1 | 1.2E-03 | -0.07 |
|  | Cytochrome c oxidase subunit VIIIa | 1.4E-03 | -0.08 |
|  | Thioredoxin | 1.7E-03 | -0.08 |
|  | Transaldolase | 1.7E-03 | 0.10 |
|  | Nadh-ubiquinone oxidoreductase AGGG subunit precursor | 2.2E-03 | 0.09 |
|  | Glycogen synthase kinase-3 alpha | 2.7E-03 | -0.07 |
|  | NADH dehydrogenase (ubiquinone) 1 alpha subcomplex 9 | 3.5E-03 | 0.06 |
|  | ADP, ATP carrier protein, fibroblast isoform | 4.1E-03 | -0.07 |
|  | Fatty acid synthase | 5.3E-03 | -0.08 |
|  | NADH dehydrogenase (ubiquinone) flavoprotein 2 | 5.3E-03 | 0.06 |
|  | Ubiquitin carboxyl-terminal hydrolase 14 | 6.5E-03 | 0.07 |
|  | Plasminogen precursor | 6.7E-03 | -0.10 |
|  | Glycerol kinase | 6.7E-03 | 0.06 |
|  | Isocitrate dehydrogenase isozyme 3 | 7.9E-03 | -0.07 |
|  | NADH-ubiquinone oxidoreductase B17 subunit | 8.2E-03 | -0.07 |
| **NEWARK** | **Myo-inositol 1-phosphate synthase A1** | 1.5E-11 | 0.27 |
|  | **Nadh-ubiquinone oxidoreductase AGGG subunit precursor** | 3.1E-06 | 0.14 |
|  | **Alanine aminotransferase** | 9.9E-06 | 0.14 |
|  | **3-hydroxyacyl-CoA dehydrogenase type II** | 1.8E-05 | 0.14 |
|  | **Adenylyl cyclase-associated protein 2** | 1.8E-05 | 0.16 |
|  | **Betaine--homocysteine S-methyltransferase** | 4.6E-05 | 0.22 |
|  | Aldehyde dehydrogenase 1 | 3.0E-04 | -0.10 |
|  | Fatty acid binding protein H6-isoform | 6.0E-04 | 0.16 |
|  | Fatty acid-binding protein, retina | 6.0E-04 | 0.13 |
|  | Thioredoxin | 1.0E-03 | 0.12 |
|  | Methylmalonate-semialdehyde dehydrogenase [acylating] | 1.5E-03 | 0.08 |
|  | NADH-ubiquinone oxidoreductase PDSW subunit | 1.5E-03 | 0.10 |
|  | Elongation factor 1-alpha | 1.8E-03 | 0.11 |
|  | Elongation factor 1-beta | 1.9E-03 | 0.13 |
|  | Troponin I, cardiac muscle | 2.4E-03 | -0.09 |
|  | Cytochrome P450 2N2 | 3.1E-03 | -0.10 |
|  | Glutathione S-transferase A | 3.3E-03 | 0.16 |
|  | Low-density lipoprotein receptor 1 precursor | 5.7E-03 | 0.10 |
|  | Cytochrome P450 8B1 sterol 12 alpha-hydrolase | 6.2E-03 | 0.10 |
|  | Hepatocyte nuclear factor 4-alpha | 6.6E-03 | -0.09 |
|  | ATP synthase H+ transporting mitochondrial F0 complex subunit d | 7.6E-03 | 0.09 |
|  | Dihydrolipoamide S-succinyltransferase | 8.4E-03 | -0.11 |
|  | Cystathionine-beta-synthase | 8.6E-03 | 0.06 |
|  | Delta-1-pyrroline-5-carboxylate dehydrogenase | 8.8E-03 | 0.09 |
|  | Dodecenoyl-CoA Delta-isomerase | 8.8E-03 | -0.09 |
|  | Glucose-6-phosphatase | 9.3E-03 | -0.12 |
|  | Protein disulfide isomerase A3 precursor | 9.4E-03 | 0.08 |
| **ER** | **Hepatocyte nuclear factor 4-alpha** | 2.3E-05 | 0.10 |
|  | Phosphomannomutase 1 | 7.4E-04 | -0.08 |
|  | Nadh-ubiquinone oxidoreductase AGGG subunit precursor | 1.0E-03 | 0.09 |
|  | Ubiquitin carboxyl-terminal hydrolase 14 | 1.8E-03 | -0.07 |
|  | Cytochrome-c oxidase chain VIc | 1.8E-03 | -0.08 |
|  | ATP synthase H+ transporting mitochondrial F0 complex subunit f isoform 2 | 2.0E-03 | -0.09 |
|  | Aldehyde dehydrogenase family 7 member A1 | 2.9E-03 | 0.09 |
|  | Fatty acid binding protein 7 brain | 3.7E-03 | -0.07 |
|  | Adenylyl cyclase-associated protein 2 | 6.4E-03 | 0.08 |
|  | Thioredoxin | 6.8E-03 | -0.13 |
|  | Cold-shock domain protein | 6.9E-03 | 0.06 |
|  | Isocitrate dehydrogenase 1 | 7.3E-03 | 0.08 |
|  | Long-chain-acyl-CoA dehydrogenase | 7.3E-03 | -0.09 |
